# Effects of 1267 nm Illumination on Microcirculation Regulatory Mechanisms

**DOI:** 10.1002/jbio.202400296

**Published:** 2024-11-26

**Authors:** Lyubov Eratova, Irina Makovik, Viktor Dremin

**Affiliations:** ^1^ R&D Center of Biomedical Photonics Orel State University Orel Russia; ^2^ College of Engineering and Physical Sciences Aston University Birmingham UK

**Keywords:** 1267 nm laser, blood flow oscillation, laser Doppler flowmetry, microcirculatory bed, singlet oxygen, wavelet analysis

## Abstract

This study explored the effects of 1267 nm laser irradiation on changes in blood flow parameters and activation of the regulatory mechanisms of the microcirculatory bed (MCB). Using laser Doppler flowmetry (LDF) technique and time‐frequency analysis of perfusion signals, changes in the MCB of 16 healthy volunteers, targeting the distal phalanx of the third finger with 1267 nm laser irradiation were evaluated. Results indicated no significant differences in perfusion between control and target measurements, likely due to blood flow redistribution caused by vessel dilation/constriction. However, differences in oscillation amplitudes in endothelial and myogenic ranges were observed, suggesting microcirculation self‐regulation. Detailed analysis revealed characteristic peaks in the endothelial range during and after irradiation, indicating endothelial mediator release. It is assumed that the identified effects may be related to the singlet oxygen generated by 1267 nm laser irradiation, which directly affects the MCB, manifesting as endothelium‐dependent vascular activity.

## Graphical abstract

The question of activation of blood flow regulatory mechanisms during laser illumination remains open to date. This study showed the effect of laser exposure on the microcirculatory bed, which appears in the form of endothelium‐dependent vascular activity.

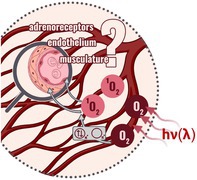

## 1 Introduction

Reactive oxygen species (ROS) are highly reactive chemical molecules. Due to their direct production by all cellular components of the vascular wall (fibroblasts, endothelial, smooth muscle and immune cells), ROS play an important role in the physiology and pathophysiology of the cardiovascular system [1, 2]. The physiological effects of ROS can be manifested both in the modulation of acute vascular functions, such as vasodilation, vasoconstriction, and vascular permeability, and the activation of long‐term vascular changes, including structural remodeling of vessel segments and the vascular channel as a whole [3, 4]. Superoxide and hydrogen peroxide have the greatest influence on these processes. However, a specific influence of singlet oxygen (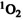) is reported.

Works [5, 6, 7] have shown that 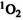 is able to affect changes in the vascular channel and blood rheological properties, manifested by blood stasis and extravasation, vascular occlusion and vascular network disconnection. The combination of these changes can lead to hypoxia and tissue destruction. Such changes are considered the dominant biological response in photodynamic therapy (PDT) using photosensitizers [7, 8].

The microcirculatory bed (MCB) is the most sensitive to photosensitizers effects due to its localization and function. MCB is the distal segment of the vascular system located between the arterial and venous sections and plays an important role in supplying tissues with nutrients and oxygen [9]. Despite the fact that 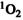 can activate irreversible changes at the level of MCB during PDT, due to the possible formation of other ROS [4], it is impossible to assess the true role of 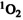 in these changes.

**FIGURE 1:**
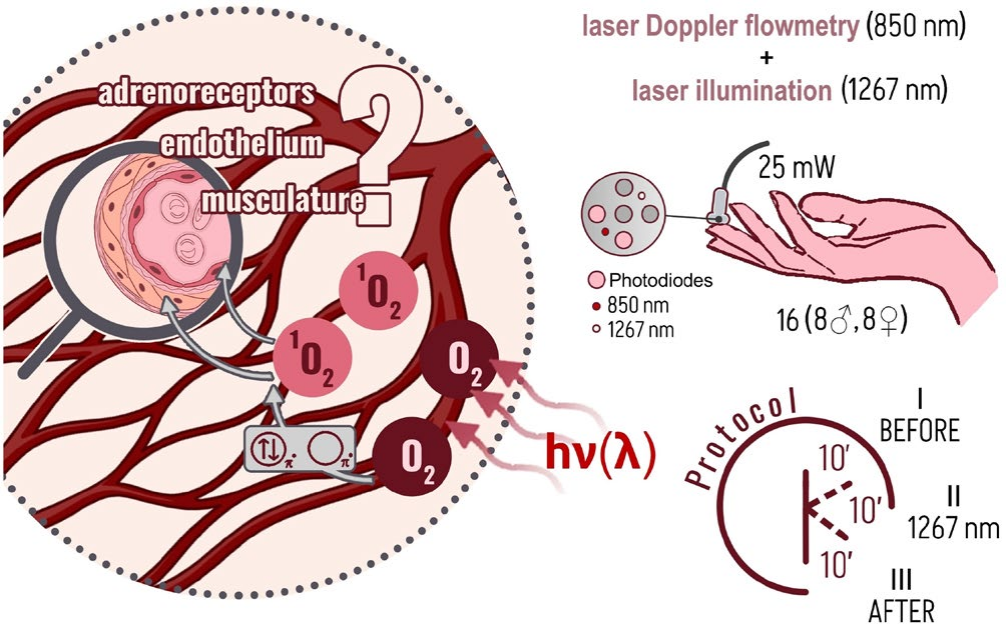
The main concept and protocol of the study. The 30‐min protocol includes continuous recording of LDF perfusion and laser irradiation for 10 min in the middle of the protocol. The representation of the fiber tip demonstrates the location of the fibers. The circles shaded in gray are the emitting and receiving fibers of the fluorescence spectroscopy channel that is not used in this study.

The possibility of realizing a different approach to 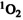 generation using laser radiation of certain wavelengths in the spectral range of 390–1300 nm [10] allowed to make a significant step in understanding the role of this ROS in the regulation of physiological functions of cells and tissues in the absence of photosensitizers. It was shown that, depending on the dose of generated 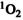 in cellular structures, both activation of mitochondrial respiration can occur [11], as well as triggering oxidative stress, destabilizing cellular metabolism [12] or cell death [13, 14, 15]. Although there is an increasing number of studies showing the effects of laser‐induced 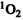 on changes in the status of the circulatory and lymphatic systems [16, 17, 18], the question of identifying the activating regulatory mechanisms and assessing their contribution (Figure 1) to total blood flow during 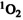 generation in the absence of photosensitizer remains open to date.

The application of a noninvasive method of laser Doppler flowmetry (LDF) with subsequent wavelet analysis of the recorded signals seems promising for solving this problem. This method is based on the analysis of optical radiation scattered from biological tissues, the frequency of which differs from the probing radiation by the value of the Doppler shift [19, 20, 21]. The LDF signal registered on the skin shows perfusion in MCB (capillaries, arterioles, venules, and arterio‐venous anastomoses) and is characterized by periodic changes. Assessment of their spectral properties in the frequency range is of great importance, taking into account the influence of regulatory mechanisms of different nature on blood flow parameters.

Although blood flow oscillations are often viewed as a source of non‐repetitive signals arising from the stochastic nature of fluctuations in the velocity of red blood cells [22], their oscillatory components correspond to specific physiological mechanisms [23]. Wavelet analysis of recorded LDF signals makes it possible to identify the contribution to total blood flow of high‐ and low‐frequency components. The high‐frequency components are mediated by the influence of heart contractions and respiratory rate, and the low‐frequency—by the myogenic mechanism of vascular tone regulation, neurogenic sympathetic vasomotor activity and vascular endothelium activation. These mechanisms are considered in detail in [22, 24, 25].

Using this approach, the effect of 1267 nm laser irradiation and the possible generation of 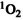 on changes in microcirculatory parameters, activating regulatory mechanisms, and their contribution to total blood flow were evaluated.

## 2 Materials and Methods

### 2.1 Volunteers Group

Sixteen conditionally healthy volunteers (8 men and 8 women) without diagnosed diseases of the circulatory system, musculoskeletal system, or connective tissue participated in the study. Volunteers with exacerbations of diseases of the cardiovascular, pulmonary, neuroendocrine systems, gastrointestinal tract, liver, kidney, blood, and any other severe chronic diseases that could affect MCB were excluded from the study. Before starting the experiments, each volunteer filled out a questionnaire containing a health survey. The mean age was 25 ± 6 years. The studies were carried out after familiarizing conditionally healthy volunteers with the study protocol and signing an informed voluntary consent. The research was approved by the local Ethical Committee of the Orel State University (protocol No. 10 of November 16, 2018).

### 2.2 Experimental Setup

A modified LDF channel of the multifunctional laser research system (SPE “LAZMA” Ltd., Russia) was used to evaluate the effect of 1267 nm‐laser irradiation on changes in the MCB parameters [26, 27, 28]. The fluorescence spectroscopy channel of this system was not used in this study. Taking into account the possible generation of 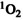 [29], the standard 1064 nm‐laser source of the LDF channel was replaced by an irradiation source with a wavelength of 850 nm [30, 31]. A custom‐built Y‐type fiber‐optic probe (3 mm ø) was used to register the LDF signal and deliver 1267 nm laser irradiation. The probing fiber of the LDF has a diameter of 6 μm, and the receiving fiber has a diameter of 400 μm. The source‐detector spacing for the LDF channel is 0.5 mm. The fiber size of the 1267 nm channel was 50 μm.

CW laser diode SM‐1267‐PM‐500 (Innolume GmbH, Germany) with a wavelength of 1267 nm was used for skin illumination. Laser diode driver SF8150‐ZIF14 (Maiman Electronics LLC, Russia) was used to supply the laser with the required electrical drive current. Recently, the effectiveness of 1267 nm irradiation to generate 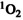 has been demonstrated using chemical traps [11, 29, 32]. Moreover, it was found that at the same doses, the exposure time factor (long irradiation time) has a more significant effect on the efficiency of 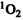 production than the laser power [29]. Given these results, *in silico* studies of thermal field distribution in biological tissues under NIR irradiation [33], as well as the size of irradiation fiber, to minimize tissue heating, the power of laser irradiation at the probe tip was chosen 25 mW, which causes local heating of tissue less than 1°C.

### 2.3 Study Protocol

The experimental measurements were conducted on the group of volunteers in two stages. The first stage included continuous recording of perfusion for 30 min with 10 min of 1267 nm laser irradiation (see Figure 1). For analysis, the records were conditionally divided into three parts: (I) before irradiation, (II) during laser exposure, and (III) after irradiation. A few days later, the same volunteers were examined for 30 min without exposure to a 1267 nm laser. Here, the recording was also divided into three parts in order to exclude the influence of changes in perfusion over time in a static posture and adequately compare with the first stage of measurements. The choice of the total recording duration and the division into parts was associated with obtaining reliable statistics for the analyzed frequencies, including low‐frequency oscillations in blood flow of endothelial origin [34].

The study was carried out approximately the same time of day (around 12:00 pm) to avoid the influence of circadian rhythms on blood circulation 2 h after a meal [26]. The volunteers also underwent a preliminary adaptation to room temperature 23°C–24°C for 15–20 min in a sitting position. The palm surface of the distal phalanx of the third finger of the right hand was chosen as the study area. The temporal variability of LDF signals on different days is known [35]. Therefore, in order to minimize this effect, each volunteer was measured three times on different days with and without laser irradiation. Thus, 96 LDF signals with a duration of 30 min each were obtained from 16 volunteers.

### 2.4 Data Processing

Based on the analysis of recorded LDF signals for each stage of the study, the index of blood microcirculation (I_*m*_), standard deviation (σ) and coefficient of variation ($k_{v}$) were evaluated. In addition, the LDF signals were subjected to continuous wavelet transform (CWT) analysis. The signals were decomposed using CWT in the form:

(1) $$W_{x}\left(s,\tau \right)=\frac{1}{\sqrt{s}}\int_{-\infty }^{\infty }x\left(t\right)\psi^{*}\left(\frac{t-\tau }{s}\right)\mathrm{dt}$$

where $x\left(t\right)$ is a target signal, τ is the time shift of the wavelet, s is the scaling factor, and the symbol 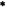 means complex conjugation. The decomposition was performed using the Morlet wavelet:

(2) $$\psi \left(t\right)=e^{2\mathrm{\pi it}}e^{-t^{2}/2\sigma^{2}}$$

It is a complex wavelet composed of a sinusoidal oscillation modulated by a Gaussian window, which provides a good balance between time and frequency localization [36, 37].

The obtained results of wavelet analysis were used to determine the maximum amplitudes of endothelial oscillations ($\sigma I_{\mathrm{me}}$) for the frequency range of 0.01–0.02 Hz, neurogenic oscillations ($\sigma I_{\mathrm{mn}}$)—0.021–0.046 Hz, myogenic oscillations ($\sigma I_{\mathrm{mn}}$)—0.047–0.145 Hz, oscillations associated with respiratory chest movements ($\sigma I_{\mathrm{mr}}$)—0.2–0.4 Hz and pulse oscillations associated with heart contractions ($\sigma I_{\mathrm{mc}}$)—0.8–1.6 Hz [22, 24, 25]. Due to the scatter of the measurements results of the oscillation amplitudes in different LDF recordings, the contribution of the amplitudes of the low‐frequency oscillations of endothelial, neurogenic, and myogenic genesis relative to the average blood flow modulation was determined for further analysis as $\sigma I_{m}/3\sigma$ (where $\sigma I_{m}$ is the amplitude of oscillations, σ is the standard deviation of the perfusion oscillations). Such normalization of the oscillation amplitudes makes it possible to exclude the influence of the study conditions since an increase or decrease in the amplitude of the oscillations and the average modulation occurs in the same direction. The value of the normalized oscillation amplitude characterizes the severity of fluctuations in a particular range in relation to the average oscillatory process.

Statistical data analysis was performed using OriginPro software (OriginLab Corp., Northampton, USA). Data are presented as a box‐whisker diagram. In each box, the central line is the median, while the edges are the 25th and 75th percentiles. The significance of the statistical differences between the samples was assessed using the Mann–Whitney *U* test. The probability was considered statistically significant at p < 0.05.

## 3 Results

Table 1 shows the results of the measurement and calculation of the main parameters of the MCB in the examined conditionally healthy volunteers.

**TABLE 1:** Results of measurement and calculation of the main parameters of the MCB.

Parameters	Laser illumination stage			Control stage		
	I	II	III	I	II	III
$I_{m}$, p.u.	21.54 ± 3.59	21.68 ± 3.48	21.34 ± 3.62	21.84 ± 2.75	21.84 ± 2.49	21.79 ± 2.42
σ, p.u.	2.04 ± 0.87	2.16 ± 0.68	2.50 ± 0.83	1.73 ± 1.04	2.01 ± 0.90	2.11 ± 0.81
k_*v*_, %	10.3 ± 5.06^c^	11.02 ± 5.63	12.26 ± 5.45^c^	8.18 ± 5.01	9.22 ± 3.8	9.69 ± 3.55
$\sigma I_{\mathrm{me}}$, p.u.	0.68 ± 0.93	0.76 ± 0.83	0.97 ± 0.91^a^, ^b^, ^c^	0.58 ± 0.51	0.70 ± 0.40	0.72 ± 0.42^a^
$\sigma I_{\mathrm{mn}}$, p.u	0.73 ± 0.36	0.91 ± 0.38^a^	1.00 ± 0.42^a^	0.67 ± 0.52	0.87 ± 0.46^a^	0.93 ± 0.41^a^
$\sigma I_{\mathrm{mm}}$, p.u.	0.59 ± 0.27	0.72 ± 0.30^a^, ^c^	0.75 ± 0.32^a^	0.51 ± 0.35	0.57 ± 0.26^a^	0.62 ± 0.27^a^
$\sigma I_{\mathrm{mr}}$, p.u.	0.18 ± 0.06	0.19 ± 0.07^c^	0.19 ± 0.06	0.16 ± 0.05	0.16 ± 0.05	0.16 ± 0.04
$\sigma I_{\mathrm{mc}}$, p.u.	0.53 ± 0.21	0.59 ± 0.24	0.57 ± 0.21	0.50 ± 0.16	0.50 ± 0.17	0.51 ± 0.17

*Note*: The data were registered in perfusion units (p.u.). I, II, III are the parts of the LDF signal before, during and after laser irradiation, respectively. For control measurements, this corresponds to a 10‐min split of the whole signal.

^a^ Confirmed statistically significant differences: II versus I and III versus I within each stage, p < 0.05.

^b^ Confirmed statistically significant differences: III versus II within each stage, p < 0.05.

^c^ Confirmed statistically significant differences for pairwise comparison of the corresponding values from each group, p < 0.05.

**FIGURE 2:**
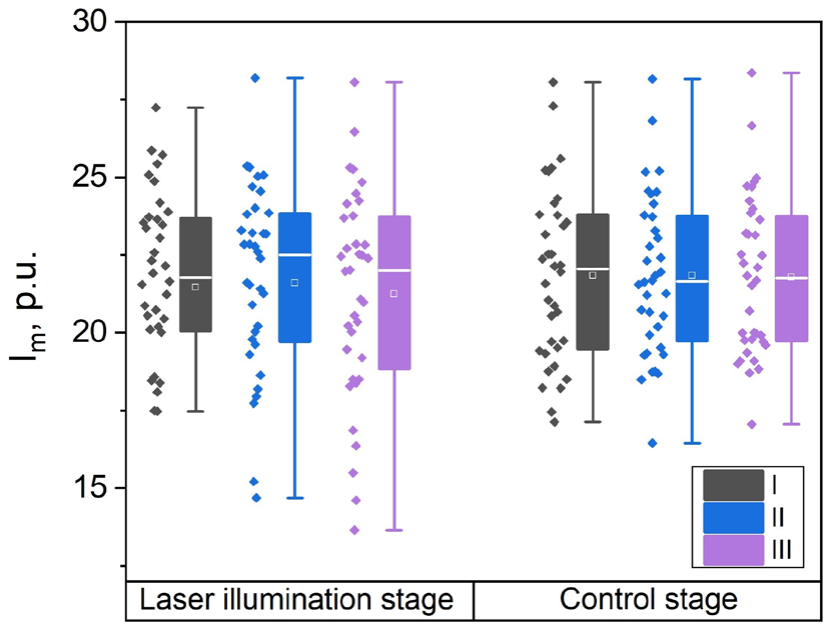
Comparison of index of blood microcirculation I_*m*_ for measurements with illumination (laser illumination stage) and without (control stage). Designations I, II, III correspond to the description in Table 1.

As can be seen from the Figure 2 and presented data in Table 1, statistically significant differences in the index of blood microcirculation (I_*m*_) and oscillation amplitudes of respiratory ($\sigma I_{\mathrm{mr}}$) and cardiac ($\sigma I_{\mathrm{mc}}$) genesis are not observed. At the same time, there are significant differences in the oscillation amplitudes of active mechanisms of regulation of MCB, indicating the activation of processes directly related to self‐regulation of MCB during laser illumination.

**FIGURE 3:**
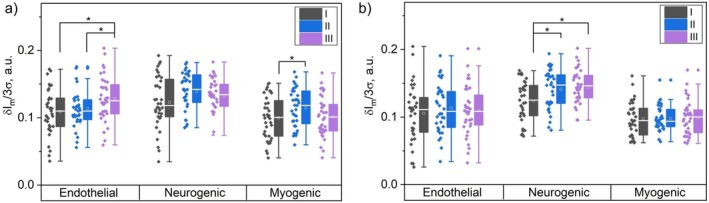
Contribution of normalized amplitudes of low‐frequency oscillations of endothelial, neurogenic, and myogenic genesis in relation to the average blood flow modulation for measurements (a) with illumination and (b) without. * Confirmed statistically significant differences, p < 0.05.

More detailed analysis of the activating mechanisms of regulation and evaluation of the contribution of amplitudes of low‐frequency oscillations of endothelial ($\sigma I_{\mathrm{me}}$), neurogenic ($\sigma I_{\mathrm{mn}}$) and myogenic ($\sigma I_{\mathrm{mm}}$) genesis to the average blood flow modulation showed differences in the activating mechanisms between measurements with illumination and control measurements (Figure 3).

As can be seen from the presented data, reliable differences in endothelial and myogenic ranges are observed due to laser illumination. In the control measurements, in the absence of laser exposure, reliable changes in the normalized amplitudes of oscillations in the neurogenic range are observed.

**FIGURE 4:**
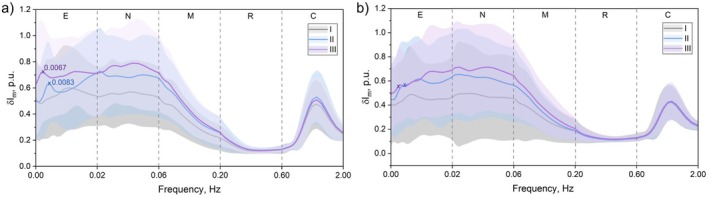
Averaged wavelet spectra of 10‐min parts (I, II, III) of LDF signals for measurements (a) with illumination and (b) without. The thick color line corresponds to the average value, the filled regions are standard deviations of each sample. E, N, M, R, C correspond endothelial, neurogenic, myogenic, respiratory and cardiac oscillations region, respectively.

Since it is impossible to judge the release of a particular endothelial vasoactive mediator based only on changes in amplitude characteristics over the entire range (0.005–0.02 Hz), a detailed analysis of the spectra for each stage of the study, obtained after wavelet transformation, was carried out in this work (0.02–2 Hz in steps of [0.02 + 0.00032 × (*n* + 1)] Hz) of LDF signals (Figure 4).

The analysis revealed a difference in the spectral characteristics of the blood flow oscillations. In the averaged wavelet spectra of LDF signals, the appearance of peaks in the endothelial range at frequencies 0.0083 Hz (II) during laser exposure and 0.0067 Hz (III) after laser exposure was observed (Figure 4a), which is different from the control measurements (Figure 4b).

## 4 Discussion

The work presents experimental data showing the effect of 1267 nm laser illumination on the state of the MCB and the mechanisms of its blood flow regulation. Although in this work the observed changes are associated with the generation of 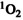, it would be correct to note that near‐infrared laser irradiation can have other photobiomodulatory effects with changes in the metabolic activity of tissues and cells [38].

Averaging I_*m*_ over the entire sample did not reveal statistically significant changes after laser exposure, at the same time, each individual subject showed different reactions of the MCB before and after irradiation (decrease or increase). Probably, blood flow redistribution occurs due to a different reaction (vasoconstriction/vasodilation) on the part of vessels of different morphology that form the MCB when exposed to laser irradiation. In that way, the changes occurring are so insignificant that they are lost in the signal with a large scatter of values, explained by the increasing microtremor of the investigated limb when recording the LDF signal with a duration of 30 min.

As for blood flow oscillations, the reliable differences observed in the endothelial and myogenic ranges in the main group and in the neurogenic range in the control group can be interpreted in different ways, taking into account the results of studies by other authors.

Considering the results of the control measurements, reliable differences of normalized oscillation amplitudes in the neurogenic range and their predominance with a decrease in the amplitudes of myogenic oscillations were established, which is associated with the predominance of the ergotropic direction of regulation of MCB and the activation of the non‐nutritive (shunt) blood flow pathways [39, 40]. It is also worth noting the dominance of sympathetic nervous activity during control measurements, which is evident from the predominance of the amplitudes of neurogenic oscillations over others. These changes are not observed at laser illumination.

In view of the role of α‐adrenoreceptors in the genesis of neurogenic oscillations in human skin, as well as the observed predominance of sympathetic nervous activity, the main mediator of which is norepinephrine (NA), it can be concluded that α‐adrenoreceptors sensitive to NA are “blocked,” presumably, by 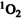, since the supposed response from the MCB is not observed in a specific time interval (II and III), as in the control study. Thus, α‐adrenoreceptors become unresponsive to NA, which is correlated with the results of the studies published earlier [41].

On the other hand, the revealed endothelial and myogenic activity may be Ca^2+^‐dependent due to the disturbance of Ca^2+^ homeostasis in the cell under the action of laser illumination and potential generation of 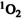 [42]. All this suggests a mechanism of endothelial activation, which is carried out directly through an increase in the cytosolic Ca^2+^ concentration bypassing adrenoreceptors (since no increase in neurogenic oscillations is observed) and subsequent relaxation of myocytes due to hyperpolarization of the endothelial cell membrane and transmission of the electrical signal through myoendothelial contacts to smooth muscle cells. However, this hypothesis requires research at the cellular level.

However, it is possible that activation of the endothelium via Ca^2+^ leads to re‐lysing of vasodilating substances, which subsequently arrive at myocytes, but this process is characterized by a longer duration, and the low‐frequency character of endothelial oscillations (about 1 oscillation per minute) does not allow us to trace how quickly the change in amplitude occurred. It is known that when considering the wavelet spectra of LDF signals, by changes in amplitudes and appearance of peaks [25, 43] in the frequency range of 0.0095–0.02 Hz, one can talk about the contribution of nitric oxide (NO) to the dilation of small arteries and large arterioles. However, the appearance of peak values is observed in a different range (0.005–0.0095 Hz) (see Figure 4a). Thus, it can be conclude that changes in MCB and activation of endothelial oscillations during laser illumination are not dependent on NO but on any other vasoactive substances. Also, other endothelial mechanisms, such as endothelium‐derived hyperpolarizing factor (EDHF), might be involved in the regulation of this frequency interval [25].

## 5 Conclusions

This study showed the direct effect of 1267 nm laser illumination on MCB. Although laser exposure does not lead to changes in perfusion, which can be associated with the suggested redistribution of blood flow at moments of dilation/constriction of vessels of different morphology, it leads to activation of endothelial and myogenic regulatory mechanisms in MCB. A more detailed analysis of changes in these spectral ranges in each of the three study stages made it possible to identify characteristic peaks in the endothelial range during and after 1267 nm tissue irradiation. These peaks are characteristic for the process of release of endothelial mediator. Therefore, the role of laser illumination in MCB changes may be related to endothelium‐dependent vascular activity.

## Conflicts of Interest

The authors declare no conflicts of interest.

## Data Availability

The data that support the findings of this study are available from the corresponding author upon reasonable request.
